# Three Novel Pathogenic Variants in Unrelated Vietnamese Patients with Cardiomyopathy

**DOI:** 10.3390/diagnostics14232709

**Published:** 2024-11-30

**Authors:** Dac Dai Tran, Nguyen Thi Kim Lien, Nguyen Van Tung, Nguyen Cong Huu, Phan Thao Nguyen, Do Anh Tien, Doan Thi Hoai Thu, Bui Quang Huy, Tran Thi Kim Oanh, Nguyen Thi Phuong Lien, Nguyen Thanh Hien, Nguyen Ngoc Lan, Le Tat Thanh, Nguyen Minh Duc, Nguyen Huy Hoang

**Affiliations:** 1 E Hospital, Ministry of Health, 89 Tran Cung Str., Cau Giay, Hanoi 100000, Vietnam; bsdai@trungtamtimmach.vn (D.D.T.); vanbandieuhanh@benhviene.com (N.C.H.); bsphanthaonguyen@gmail.com (P.T.N.); doanhtien@trungtamtimmach.vn (D.A.T.); bsthu@trungtamtimmach.vn (D.T.H.T.); dr.quanghuytttm@gmail.com (B.Q.H.); ttkoanh1287@gmail.com (T.T.K.O.); ljenxik@gmail.com (N.T.P.L.); 2 Institute of Genome Research, Vietnam Academy of Science and Technology, 18-Hoang Quoc Viet Str., Cau Giay, Hanoi 100000, Vietnam; ntkimlienibt@gmail.com (N.T.K.L.); tungnv53@gmail.com (N.V.T.); hienmiu271095.vnu@gmail.com (N.T.H.); ngoclana11108@yahoo.com (N.N.L.); thanh.biotech@gmail.com (L.T.T.); ducnguyen24vn@gmail.com (N.M.D.); 3 Faculty of Biotechnology, Graduate University of Science and Technology, Vietnam Academy of Science and Technology, Cau Giay, Hanoi 100000, Vietnam; 4 Center for Gene and Protein Research, Hanoi Medical University, 1st Ton That Tung Str., Dong Da, Hanoi 100000, Vietnam; 5 National Research Center for Medicinal Plant Germplasm & Breeding, National Institute of Medicinal Materials, Thanh Tri, Hanoi 100000, Vietnam

**Keywords:** cardiomyopathy, dilated cardiomyopathy (DCM), hypertrophic cardiomyopathy (HCM), pathogenic variants, Vietnamese patients, whole-exome sequencing (WES)

## Abstract

**Background**: Cardiomyopathy, including dilated cardiomyopathy (DCM) and hypertrophic cardiomyopathy (HCM), is a major cause of heart failure (HF) and a leading indication for heart transplantation. Of these patients, 20–50% have a genetic cause, so understanding the genetic basis of cardiomyopathy will provide knowledge about the pathogenesis of the disease for diagnosis, treatment, prevention, and genetic counseling for families. **Methods**: This study collected nine patients from different Vietnamese families for genetic analysis at The Cardiovascular Center, E Hospital, Hanoi, Vietnam. The patients were diagnosed with cardiomyopathy based on clinical symptoms. Whole-exome sequencing (WES) was performed in the Vietnamese patients to identify variants associated with cardiomyopathy, and the Sanger sequencing method was used to validate the variants in the patients’ families. The influence of the variants was predicted using in silico analysis tools. **Results**: Nine heterozygous variants were detected as a cause of disease in the patients, three of which were novel variants, including c.284C>G, p.Pro95Arg in the *MYL2* gene, c.2356A>G, p.Thr786Ala in the *MYH7* gene, and c.1223T>A, p.Leu408Gln in the *DES* gene. Two other variants were pathogenic variants (c.602T>C, p.Ile201Thr in the *MYH7* gene and c.1391G>C, p.Gly464Ala in the *PTPN11* gene), and four were variants of uncertain significance in the *ACTA2*, *ANK2*, *MYOZ2*, and *PRKAG2* genes. The results of the in silico prediction software showed that the identified variants were pathogenic and responsible for the patients’ DCM. **Conclusions**: Our results contribute to the understanding of cardiomyopathy pathogenesis and provide a basis for diagnosis, treatment, prevention, and genetic counseling.

## 1. Introduction

Cardiomyopathies are considered as myocardial diseases with abnormal structure or function of the heart. Cardiomyopathy is a heterogeneous disease due to abnormalities in the function of the heart muscle, and is divided into dilated cardiomyopathy (DCM), hypertrophic cardiomyopathy (HCM), arrhythmogenic cardiomyopathy (ACM), and left ventricular non-compaction (LVNC) cardiomyopathy [1].

DCM has a prevalence ranging from 1:250 to 1:2500 and is the most common cause of heart failure (HF) leading to heart transplantation worldwide [2,3]. DCM is characterized by dilatation, leading to a loss of contractility and systolic dysfunction in one or both ventricles, but this usually takes the form of left ventricular (LV) dilatation in the absence of hypertension, valvular heart disease, or coronary artery disease [4,5]. DCM is found in 60% of cases of cardiomyopathy in children, with a peak incidence in the first year of life [6]. DCM seriously affects health and often leads to HF or sudden cardiac death in patients [4]. The imaging diagnosis of DCM via echocardiogram is defined by the presence of fractional shortening <25%, left ventricular ejection fraction (LVEF) <45%, and left ventricular end-diastolic diameter >2.7 cm/m^2^ or >117% predicted [7,8]. Among DCM patients, an estimated 20–50% have a genetic cause [2,9], and emerging data suggest that genotype has an important impact on prognosis and treatment [9]. Echocardiography is an important imaging test in the evaluation of patients with DCM. It provides information that helps in the diagnosis, risk stratification, treatment guidance, and screening of family members [10]. In addition, genetic diagnosis is of increasing importance as it can help predict prognosis, treatment, and prevention. Genetic testing in DCM cases has provided insights into the genetic basis of DCM and improved the knowledge on pathogenesis and genetic counseling for families. DCM is characterized by the inheritance of an autosomal dominant with incomplete penetrance leading to variable expressions of disease in terms of symptom severity and complication risk [2,11]. To date, more than 250 genes have been reported to be associated with the development of DCM, the majority of which are genes encodings proteins important for mitochondrial function and cellular integrity [12], including the genes coding for sarcomeric proteins, Z-disk-associated proteins, cytoskeletal proteins, and nuclear envelope proteins [2,8,11,13,14].

HCM has an estimated prevalence of 1:500 and is characterized by LV wall thickening with an increased number of cardiomyocytes [15]. HCM is usually inherited in an autosomal dominant pattern with variable penetrance [16]. Clinical presentation can vary from patient to patient, even within the same family, ranging from asymptomatic to severe symptoms such as HF or sudden cardiac death (SCD) [17]. The disease can remain clinically asymptomatic for many years, but is also the most common cause of SCD in young people [18]. More than 50 genes have been associated with HCM [19], and most of these genes involve encoded proteins of the sarcomere; the contractile unit of cardiomyocytes [20] explains the cause of disease in 35–60% of HCM patients.

In this study, whole-exome sequencing (WES) was performed to detect variants in disease-related genes in Vietnamese patients with cardiomyopathy. Information on variants in patients will provide a scientific insight on pathogenesis for physicians, resulting in definitive diagnosis, treatment, prevention, and genetic counseling for patients.

## 2. Materials and Methods

### 2.1. Patients and Clinical Information

Patients were recruited from nine unrelated families at the Cardiovascular Center, E Hospital, Hanoi, Vietnam. The patients included five girls and four boys, aged 1 to 10 years (mean age 5.33 years), with no family history of cardiomyopathy. Patients had no history of congenital heart disease detected before birth. Based on Doppler ultrasound results and the detailed clinical information listed in Table 1, eight patients were identified as having DCM and one patient as having HCM.

### 2.2. Exome Sequencing

DNA was extracted from patient blood samples using the Qiagen DNA Blood Mini kit (QIAGEN, Hilden, Germany). WES was performed using the SureSelect Human All Exon V7 kit with a library enriched using the SureselectXT Reagent kit (Agilent, Santa Clara, CA, USA). Data were aligned and compared with the human reference genome sequence version Hg19 using BWA 0.7.17 software (https://mybiosoftware.com/bwa-burrows-wheeler-aligner-short-long-reads.html, access date on 1 October 2024). Picard v2.18.2 software (https://github.com/broadinstitute/picard/tree/2.18.2, access date on 1 October 2024) and Genome Analysis Toolkit v3.4 software (https://gatk.broadinstitute.org/hc/en-us, access date on 1 October 2024) were used to align and detect variants. The impact of the variants on protein function was determined using SnpEff v4.1 software (https://github.com/databricks/SnpEff/blob/master/html, access date on 1 October 2024). Potentially pathogenic variants in patients were screened against published genes associated with DCM (Table S1) and HCM (Table S2). Pathogenic and rare variants with low frequency in the population (MAF < 0.001) were screened. Novel mutations were checked against dbSNP (http://ncbi.nlm.nih.gov/snp/, access date on 1 October 2024), the 1000 Genomes Project (https://www.internationalgenome.org/1000-genomes-browsers/index.html, access date on 1 October 2024), the Exome Sequencing Project (https://hgdownload.soe.ucsc.edu/gbdb/hg19/evs/, access date on 1 October 2024), the ExAC database (https://exac.broadinstitute.org, access date on 1 October 2024), and in-house databases (*n* = 300).

### 2.3. Sanger Sequencing

Sanger sequencing was performed to confirm variants identified by WES in the sample against samples from the patients’ families. Primer pairs for polymerase chain reaction and Sanger sequencing were designed using Priemer3 Plus software (https://www.primer3plus.com/index.html, access date on 1 October 2024) and synthesized by PHUSA Biochem Company (Cantho, Vietnam). Purified PCR products were sequenced directly on an ABI 3500 sequencer (Applied Biosystems, Foster City, CA, USA). The sequencing results were analyzed and compared with reference sequences in the NCBI to identify variants using BioEdit 7.2 software (https://bioedit.software.informer.com/7.2/, access date on 1 October 2024).

### 2.4. Prediction Analysis

The influence of any nucleotide changes was evaluated with the following in silico analysis tools: CADD (https://cadd.bihealth.org/snv, access date on 1 October 2024), Fathmm (http://fathmm.biocompute.org.uk/inherited.html, access date on 1 October 2024), M-CAP (http://bejerano.stanford.edu/mcap/, access date on 1 October 2024), Meta (http://www.hrc.es/investigacion/metadisc_en.htm, access date on 1 October 2024), Mutation assessor (http://mutationassessor.org/r3/, access date on 1 October 2024), Mutation Taster (https://www.mutationtaster.org/, access date on 1 October 2024), PolyPhen 2 (http://genetics.bwh.harvard.edu/pph2/, access date on 1 October 2024), PROVEAN (http://provean.jcvi.org/index.php, access date on 1 October 2024), SIFT (https://sift.bii.a-star.edu.sg/www/SIFT_seq_submit2.html, access date on 1 October 2024), and SNP&GO (https://snps-and-go.biocomp.unibo.it/snps-and-go/, access date on 1 October 2024).

A comparison of the protein sequences was performed using Clustal X2.1 software (https://clustalx.software.informer.com/2.1/, access date on 1 October 2024) based on the protein sequences of the following species: *Homo sapiens*, *Bos indicus*, *Canis lupus*, *Cricetulus griseus*, *Equus caballus*, *Gallus gallus*, *Mus musculus*, *Rattus norvegicus*, and *Sus scrofa*. To evaluate the effect of variants on protein structure, the three-dimensional structure of the wild type and mutant type was constructed using Swiss-PDB Viewer v4.1 (https://swiss-pdb-viewer.software.informer.com/4.1/, access date on 1 October 2024) with the PDB templates of P10916 (for MYL2), Q06124 (for PTPN11), P35609 (for ACTA2), Q9NPC6 (for MYOZ2), P12883 (for MYH7), and P17661 (for DES).

## 3. Results

**Figure 1:**
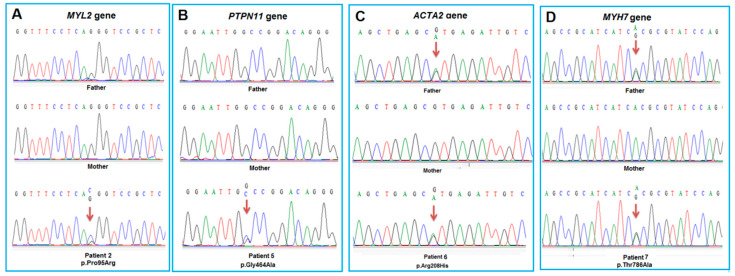
Sanger sequencing result confirms p.Pro95Arg in the *MYL2* gene in family members of patient P2 (**A**); p.Gly464Ala variant in the *PTPN11* gene in family members of patient P5 (**B**); p.Arg208His in the *ACTA2* gene in family members of patient P6 (**C**); and p.Thr786Ala in the *MYH7* gene in family members of patient P7 (**D**).

In our study, nine patients from unrelated Vietnamese families were collected for investigation. Patients had no family history of cardiomyopathy and were diagnosed based on echocardiography (Table 1). The earliest-diagnosed patient was diagnosed at 12 days of age (patient P8) and the latest at nine years of age (patient P1). Among the studied patients, eight were diagnosed with DCM and one with HCM. WES was performed on patient samples and nine heterozygous variants were identified in the following genes: *ANK2* (c.9161C>G, p.Ala3054Gly), *MYL2* (c.284C>G, p.Pro95Arg), *MYOZ2* (c.326C>G, p.Pro109Arg), *MYH7* (c.602T>C, p.Ile201Thr and c.2356A>G, p.Thr786Ala), *PTPN11* (c.1391G>C, p.Gly464Ala), *ACTA2* (c.623G>A, p.Arg208His), *PRKAG2* (c.83A>C, p.His28Pro), and *DES* (c.1223T>A, p.Leu408Gln) (Table 2). Three novel variants (c.284C>G, p.Pro95Arg in the *MYL2* gene, c.2356A>G, p.Thr786Ala in the *MYH7* gene, and c.1223T>A, p.Leu408Gln in the *DES* gene) were found in the patients. The variants in the *MYH7* gene (c.602T>C, p.Ile201Thr, rs397516258) and in the *PTPN11* gene (c.1391G>C, p.Gly464Ala, rs121918469) are identified as a pathogenic variants on the ClinVar database (under accession numbers VCV000043093.22 and VCV000013343.48, respectively). Four rare variants (with MAF<0.001) in the *ANK2*, *MYOZ2*, *ACTA2*, and *PRKAG2* genes have been reported in dbSNP data under accession numbers rs139007578, rs546999011, rs1057521703, and rs138051386, respectively, but are not evaluated on the ClinVar database. The Sanger sequencing results showed that the variants were either *de novo* in patients P2 and P5 or inherited from the asymptomatic father in patients P6 and P7 (Figure 1).

The evaluation results for the variants using the prediction software (Table 3) showed that the novel variants were pathogenic, so these variants were considered to be the cause of disease in the patients. For the variants not yet evaluated in the ClinVar database, the variants in the *ANK2* and *ACTA2* genes were also considered pathogenic by the software. Variant c.326C>G, p.Pro109Arg in the *MYOZ2* gene is considered pathogenic by FATHMM MKL, MCAP, Mutation Taster, PolyPhen 2, and CADD, but as neutral by FATHMM, Meta, PROVEAN, SIFT, and SNP&GO. Likewise, variant c.83A>C, p.His28Pro in the *PRKAG2* gene was evaluated as pathogenic by almost all software (FATHMM, MCAP, Mutation taster, SIFT, and CADD), but on the contrary as neutral by Meta and PROVEAN, and benign by PolyPhen2.

**Figure 2:**
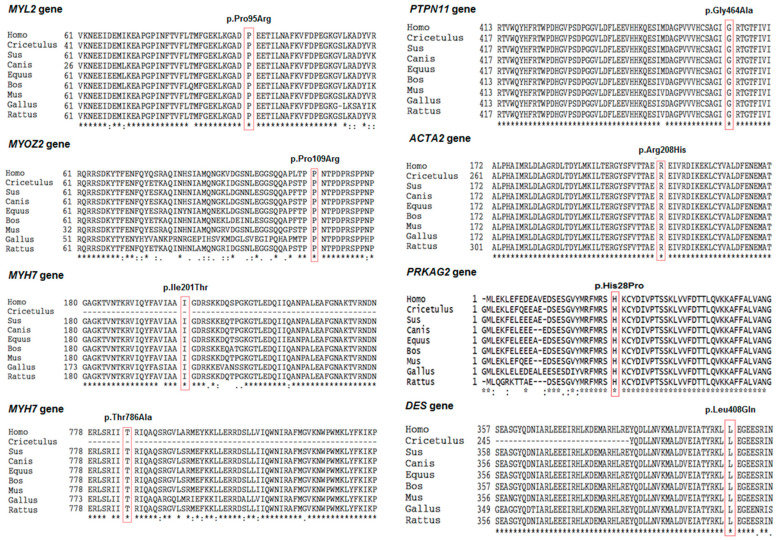
Multiple alignment of the proteins from human and other species by Clustal-X2. Multiple alignments of the MYL2, MYOZ2, MYH7, PTPN11, ACTA2, PRKAG2, and DES proteins at the positions of variants based on amino acid sequences from different species, including *Homo sapiens*, *Cricetulus griseus*, *Sus scrofa*, *Canis lupus*, *Equus caballus*, *Bos taurus*, *Mus musculus*, *Gallus gallus*, and *Rattus norvegicus*.

**Figure 3:**
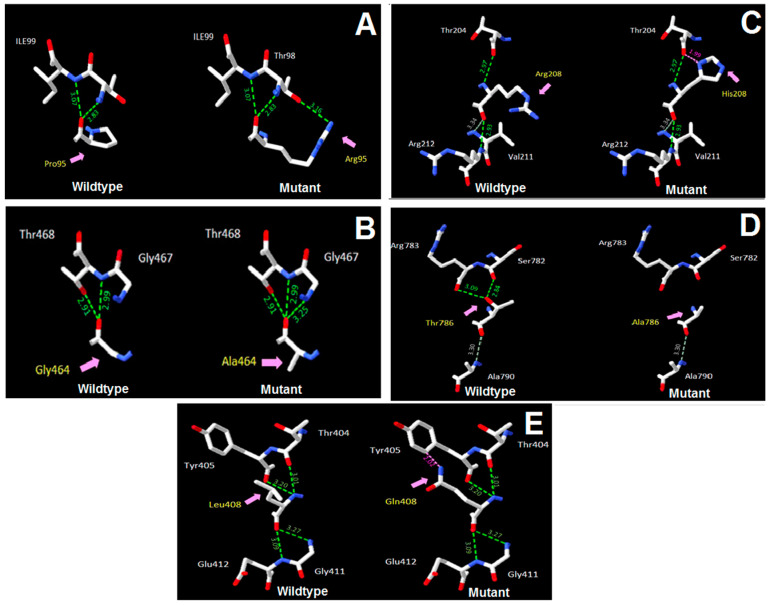
Three-dimensional structure of the proteins predicted by Swiss-Pdb Viewer. (**A**) Mutant type of MYL2 protein with the formation of one more strong H bond (in green color) between Arg95 and Thr98. (**B**) Mutant type of PTPN11 protein with the formation of one more strong H bond (in green color) between Ala464 and Gly467. (**C**) Mutant type of ACTA2 protein with the formation of one more weak H bond (in pink color) between His208 and Thr204. (**D**) Mutant type of MYH7 protein with the loss of two strong H bonds (in green color) between Ala786 with Ser782 and Arg783. (**E**) Mutant type of DES protein with the formation of one more weak H bond (in pink color) between Gln408 and Tyr405.

A similarity analysis of the protein sequences also showed that the variants were located in regions that were highly conserved between species (Figure 2). Therefore, changes in amino acids in these regions can lead to changes in the structure and function of the protein. Moreover, 3D structure analysis showed that the substitution of the amino acid Proline with Arginine at position 95 in the MYL2 protein also resulted in the formation of an additional hydrogen bond between this amino acid and Threonine at position 98 (Figure 3A). Figure 3B showed that the replacement of the amino acid Glycine with Alanine at position 464 in the PTPN11 protein resulted in the formation of an additional hydrogen bond between this amino acid and Glycine at position 467. Meanwhile, the replacement of the amino acid Arginine with Histidine at position 208 in the ACTA2 protein only resulted in the formation of an additional weak bond between this amino acid and the amino acid Threonine at position 204 (Figure 3C). This was also observed in the case of the substitution of Leucine with Glutamine at position 408 in the DES protein, which resulted in the formation of a weak bond between this amino acid and Tyrosine at position 405 (Figure 3E). In contrast, substituting the amino acid Threonine with Alanine at position 786 in the MYH7 protein resulted in the loss of two hydrogen bonds between this amino acid and the amino acids Arginine and Serine at positions 783 and 782, respectively (Figure 3D).

## 4. Discussion

In this study, the patients were diagnosed with DCM or HCM by cardiac Doppler ultrasound. The patients included five girls and four boys, aged from 1 to 10 years old (mean age was 5.33 years old), and had no family history of cardiomyopathy. WES identified nine heterozygous variants in related genes (*ANK2*, *MYL2*, *MYOZ2*, *MYH7*, *PTPN11*, *ACTA2*, *PRKAG2*, and *DES*) that were the cause of disease in patients. Among them, two variants (c.602T>C, p.Ile201Thr in the *MYH7* gene (patient P4) and c.1391G>C, p.Gly464Ala in the *PTPN11* gene (patient P5)) are reported as pathogenic in the ClinVar database. The results of assessing the harmfulness of the found variants (Table 3) showed that three novel variants (*MYL2* (c.284C>G, p.Pro95Arg) (patient P2), *MYH7* (c.2356A>G, p.Thr786Ala) (patient P7), and *DES* (c.1223T>A, p.Leu408Gln) (patient P9)) were pathogenic variants. Of the five unevaluated variants, two variants (*ANK2* (c.0161C>G, p.Ala3054Gly) (patient P1) and *ACTA2* (c.623G>A, p.Arg208His) (patient P6)) were evaluated as pathogenic variants by in silico prediction software. Two variants (*MYOZ2* (c.326C>G, p.Pro109Arg) (patient P3) and *PRKAG2* (c.83A>C, p.His28Pro) (patient P8)) were also considered as pathogenic by most of the software.

The role of the *ANK2* gene has also been reported in patients with DCM [21]. However, variants in the *MYOZ2* and *PRKAG2* genes are commonly reported in patients with HCM [22,23]. Several recent studies have shown that variants in the *MYL2* gene are also detected in patients with DCM [24]. The *MYL2*, *MYOZ2*, and *PRKAG2* genes were also included in a 111-gene panel to screen for variants associated with DCM in the study by McNally and Mestroni [8]. It is noteworthy that the *ACTA2* gene encodes specific α-smooth muscle actin, which is an isoform of vascular smooth muscle actin. This mutation mainly leads to aortic pathologies, but it also presents multisystemic smooth muscle dysfunction [25]. The *ACTA2* variant is the main cause of familial thoracic aortic aneurysms and dissection [26]. However, a variant (c.623G>A, p.Arg208His) in the *ACTA2* gene was found in one patient in our study. This result may be due to variations in the *ACTA2* gene that present multisystemic smooth muscle dysfunction, and these findings in our study may provide new insights into the role of these genes in the pathogenesis of DCM.

Molecular and genetic studies have established that at least 40% of DCM cases are due to genetic variants [8,26]. Various genes, particularly genes for myocyte membrane factors, contractile factors, Z-disc factors, myofibrillar factors, and nuclear membrane factors, can contain pathogenic variants contributing to the pathogenesis of hereditary DCM. These effects have subsequently been demonstrated in various DCM-causing variants in actin, troponin, and tropomyosin, and appear to be a general property of muscle proteins [27]. Most DCM genes are inherited in an autosomal dominant manner and have incomplete penetrance [2]. Notably, genetic causes are identified more commonly in pediatric patients than in adults (54% versus 27%) [28]. DCM is characterized by different disease manifestations in terms of time of disease onset, severity of symptoms, and risk of complications, which creates disease heterogeneity in patients [26]. Although there is a correlation between genotype and phenotype, there are large differences between people carrying the same genetic variant, even within families [29]. A significant proportion of DCM patients (20–38%) may have an oligogenic basis due to carrying multiple rare variants from unlinked loci with varying penetrance, resulting in a similar phenotype [14]. In recent years, studies have shown that the incomplete penetrance of different variants leads to differences in clinical manifestations in patients, especially in cardiomyopathy [30,31,32]. Genetic testing for cardiomyopathy has a diagnostic yield of up to 40%, but is challenging due to genetic heterogeneity, variable expressivity, and incomplete penetrance. Genotype-positive and phenotype-negative relatives are candidates for serial evaluation, with frequency varying with age [33]. McGurk et al. [34] pointed out that understanding the penetrance of pathogenic variants as secondary findings (SFs) will be important as genetic testing becomes more widespread. The authors estimated that the penetrance in late adulthood of rare pathogenic variants (23% for HCM and 35% for DCM) and likely pathogenic variants (7% for HCM, 10% for DCM) is significant in dominant cardiomyopathy. The penetrance of variants in genes associated with HCM is significantly higher for loss-of-function or rare variants and higher in males than females. Similarly, Cabrera-Romero et al. [35] evaluated penetrance and disease risk in 779 patients who carried variants of *TTN* but did not present with DCM. The authors also point out that the relationship between age and incomplete penetrance in disease expression leads to major challenges in disease management. This may also explain the cause of disease in the patients in our study, especially the two cases (patient P6 and P7) where the Sanger sequencing results showed that the patients’ fathers also carried the variant but did not show symptoms of the disease.

In our study, patient P5 was diagnosed with HCM, and genetic analysis showed that the patient carried a pathogenic variant in the *PTPN11* gene. Variants in the *PTPN11* gene, which encodes protein–tyrosine phosphatase, nonreceptor type 11, have also been reported to cause HCM in patients [36]. Although HCM is generally a relatively benign disease that may remain asymptomatic for many years, HCM carries a high risk of leading to SCD, with a leading mortality rate in the young [37]. The most devastating complication of HCM is that it is the cause of SCD in young people when the disease first appears [38]. A current challenge is the incomplete understanding of genetic variations in genes associated with HCM; thus, genetic testing is of great significance in identifying at-risk individuals early before clinical disease onset. Understanding the pathogenesis of HCM allows for the development of treatments, possible prevention, clinical management, and genetic counseling for patients.

## 5. Conclusions

In this study, nine pathogenic variants, including three novel variants, were identified in the genes *ANK2*, *MYL2*, *MYOZ2*, *MYH7*, *PTPN11*, *ACTA2*, *PRKAG2*, and *DES*, which were considered the cause of disease in patients with DCM and HCM. Our results contribute to understanding the pathogenesis of cardiomyopathy and provide a basis for diagnosis, prevention, and the development of treatments.

## Figures and Tables

**Table 1:** Clinical information of patients in the study.

Patient	Sex/Age	Rapid Heart Rate (bpm)	Sp0_2_ (%)	EF (%)	Dd (mm)	NT-ProBNP (pg/mL)	Right Ventricular TAPSE (mm)	Diagnosed
P1 TM5	Female 9 y	60	94	18	59	7260	20	DCM at 9 years old
P2 TM6	Female 2 y	178	97	30	37	-	-	DCM at 2 months of age
P3 TM13	Male 8 y	150	97	30	32	-	12	DCM at 5 years old
P4 TM14	Female 3 y	132	98	16	58	854	-	DCM at 8 months of age
P5 TM18	Female 2 y	130	98	90	17	-	-	HCM at 20 days old
P6 TM22	Female 4 y	120	98	23	46	-	-	DCM at 1 year old
P7 TM26	Male 9 y	100	98	23	54	9496	-	DCM at 6 years old
P8 TM29	Male 1 y	132	98	85	29	-	-	DCM at 12 days old
P9 TM38	Male 10 y	100	98	14	57	28,835	8	DCM at 8 years old

**Table 2:** Variants identified in the patients.

Patient	Gene	cDNA	Protein	dbSNP/MAF	ClinVar
P1	*ANK2* (AD)	c.9161C>G	p.Ala3054Gly	rs139007578 0.000001	Uncertain significance
P2	*MYL2* (AD)	c.284C>G	p.Pro95Arg	novel	
P3	*MYOZ2* (AD)	c.326C>G	p.Pro109Arg	rs546999011 0.000199681	Uncertain significance
P4	*MYH7* (AD)	c.602T>C	p.Ile201Thr	rs397516258	Pathogenic
P5	*PTPN11* (AD)	c.1391G>C	p.Gly464Ala	rs121918469	Pathogenic
P6	*ACTA2* (AD)	c.623G>A	p.Arg208His	rs1057521703 0.000001	Uncertain significance
P7	*MYH7* (AD)	c.2356A>G	p.Thr786Ala	novel	
P8	*PRKAG2* (AD)	c.83A>C	p.His28Pro	rs138051386 0.00219649	Uncertain significance
P9	*DES* (AD)	c.1223T>A	p.Leu408Gln	novel	

**Table 3:** Prediction results using in silico prediction software.

Gene/Variants	*ANK2* c.9161C>G, p.Ala3054Gly	*MYL2* c.284C>G, p.Pro95Arg	*MYOZ2* c.326C>G, p.Pro109Arg	*ACTA2* c.623G>A, p.Arg208His	*MYH7* c.2356A>G, p.Thr786Ala	*PRKAG2* c.83A>C, p.His28Pro	*DES* c.1223T>A, p.Leu408Gln
dbSNP	rs139007578	Novel	rs546999011	rs1057521703	Novel	rs138051386	Novel
FATHMM Score/Prediction	−4.18 Damaging	−1.20 Tolerated	−0.02 Tolerated	−3.50 Damaging	−3.14 Damaging	−2.30 Damaging	−3.82 Damaging
FATHMM MKL Score/Prediction	0.911 Damaging	0.964 Damaging	0.994 Damaging	0.987 Damaging	0.979 Damaging	0.616 Damaging	0.985 Damaging
MCAP Score/Prediction	0.393 Damaging	-	0.060 Damaging	0.558 Damaging	0.690 Damaging	0.061 Damaging	0.298 Damaging
Meta Score/Prediction	1.061 Damaging	-	−0.171 Tolerated	0.989 Damaging	0.526 Damaging	−0.639 Tolerated	1.086 Damaging
Meta LR Score/Prediction	0.939 Damaging	-	0.446 Tolerated	0.909 Damaging	0.764 Damaging	0.433 Tolerated	0.959 Damaging
Mutation assess Score/Prediction	2.515 Medium	-	3.205 Medium	2.030 Medium	3.160 Medium	-	4.920 High
Mutation taster Score/Prediction	0.863 Damaging	Benign	1.000 Damaging	1.000 Damaging	0.999 Damaging	0.999 Damaging	0.999 Damaging
PolyPhen 2 Score/Prediction	1.000 Damaging	0.993 Damaging	0.988 Damaging	0.992 Damaging	0.096 Benign	0.118 Benign	0.999 Damaging
PROVEAN Score/Prediction	−1.68 Neutral	−6.38 Damaging	−2.100 Neutral	−3.550 Damaging	−3.760 Damaging	0.760 Neutral	−5.110 Damaging
SIFT Score/Prediction	0.017 Damaging	0.000 Damaging	0.063 Tolerated	-	0.001 Damaging	0.044 Damaging	0.000 Damaging
SNP&GO Score/Prediction	-	R10 Disease	R5 Neutral	R2 Neutral	R10 Disease	-	R9 Disease
CADD Score/Prediction	24.8 Damaging	26.1 Damaging	27.6 Damaging	33.0 Damaging	23.8 Damaging	16.9 Damaging	31.0 Damaging

## Data Availability

Data is contained within the article or Supplementary Materials.

## Supplementary Materials

The following supporting information can be downloaded at: https://www.mdpi.com/article/10.3390/diagnostics14232709/s1, Table S1: List of published genes associated with dilated cardiomyopathy was used for screening variants in the study; Table S2: List of published genes associated with hypertrophic cardiomyopathy was used for screening variants in the study.
