# Supplementary material for: Three Novel Pathogenic Variants in Unrelated Vietnamese Patients with Cardiomyopathy
Source: Diagnostics (Basel). 2024 Nov 30;14(23):2709. doi: 10.3390/diagnostics14232709 (PMC11640685; doi:10.3390/diagnostics14232709)
Supplement: Supplementary file 1 [file diagnostics-14-02709-s001.zip › diagnostics-3293743-supplementary.pdf]

Table S1. List of published genes associated with dilated cardiomyopathy was used for screening variants in the study

Gene	Protein	Phenotype	Inheritance	OMIM/Chr
ABCC9	ATP binding cassette C9	DCM	AD	601439/12p12
ACTA2	Actin alpha 2	DCM	AD	102620/10q23.31
ACTC1	Actin alpha cardiac	DCM/HCM	AD	102540/15q14
ACTN2	Actinin alpha 2	DCM	AD	102573/1q43
ANK2	Ankyrin 2	DCM	AD	106410/4q25
ANKRD1	Ankyrin repeat domain containing protein	DCM/HCM	AD	609599/10q23.31
BAG3	Bcl2-associated athanogene 3	DCM	AD	603883/10q26
CRYAB	Crystallin-alpha B	DCM/HCM	AD	123590/11q23
CSRP3/ MLP	Cystein- and glycine-rich protein 3	DCM/HCM	AD	600824/11p15.1
CTF1	Cardiotrophin 1	DCM	AD	600435/16p12.1
DES	Desmin	DCM/RCM	AD	125660/2q35
DMD	Dystrophin	DCM	X-linked	300377/Xp21.2
DSG2	Desmoglein 2	DCM	AD	125671/18q12.1
DOLK	Dolichol kinase	DCM	AR	610746/9q34.11
DSP	Desmoplakin	DCM	AD	125647/6p24.3
DTNA	Dystrobrevin alpha	DCM	AD	601239/18q12.1
EMD	Emerin	DCM	X-linked	300384/Xq28
EPG5	Ectopic P-granules autophagy protein 5	DCM	AR	615068/18q12.3
EYA4	Eyes-absent 4	DCM	AD	603550/6p23.2
FHL2	Four and a half Lim domains 2	DCM	AD	602633/2q12.2
FKTN	Fukutin	DCM	X-linked	611615/9q31.2
FLNC	Filamin C	DCM	AD	102565/7q32.1
GATAD1	GATA zinc finger domain-containing protein 1	DCM	AR	614518/7q21.2
ILK	Integrin linked kinase	DCM	AD	602366/11q15.4
JUP	Plakoglobin	DCM	AR/AD	173325/17q21.2
LAMP2	Lysosome associated membrane protein 2	DCM	X-linked	309060/Xq24
LAMA4	Laminin alpha 4	DCM	AD	600133/6q21
LDB3	Lim domain binding 3	DCM	AD	605906/10q23
LMNA	Lamin A/C	DCM	AD	150330/1q22
MURC	Muscle-restricted coiled-coil	DCM/HCM	AD	617714/9q31.1
MYBPC3	Myosin binding protein 3	DCM/HCM	AD	600958/11p11.2
MYH2	Myosin heavy chain 2	DCM/HCM	AD/AR	605637/17p13.1
MYH6	Myosin heavy chain 6	DCM/HCM	AD	160710/14q11.2
MYH7	Myosin heavy chain 7	DCM/HCM/RCM	AD	160760/14q11.2
MYL2	Myosin regulatory light chain	DCM/HCM	AD/AR	160781/12q24.11
MYL3	Myosin regulatory light chain	DCM/HCM	AD	160790/3p21.31
MYOZ2	Myozenin 2	DCM/HCM	AD	605602/4q26
MYPN	Myopalladin	DCM/HCM/RCM	AD	608517/10q21
NEBL	Nebulette	DCM	AD	605491/10p12.11

NEXN	Nexilin	DCM/HCM	AD	613121/1p43
NKX2-5	NK2 Homeobox 5	DCM	AD	600584/5q35.1
PKP2	Plakophillin 2	DCM	AD	602861/12p11.21
PLB	Phospholamban	DCM	AD	609909/2p23.2
PLN	Phospholamban	DCM	AR	172405/6q22.11
PRDM16	PR/SET Domain 16	DCM	AD	60557/1p36.32
PRKAG2	Protein kinase 2 AMP-activated Gamma 2	DCM	AD	602743/7q36.1
PSEN1	Presenilin 1	DCM	AD	104311/14q24.2
PSEN2	Presenilin 2	DCM	AD	300377/1q42.13
RBM20	RNA-binding motif protein 20	DCM	AD	613171/10q25.2
SCN5A	Sodium channel voltage-gated 5A	DCM	AD	600163/3p22.2
SDHA	Flavoprotein	DCM	AR	600857/5p15.33
SGCD	Sarcoglycan-delta	DCM	AD	601411/5q33.2
SLC40A1	Solute carrier family 40 member 1	DCM	AD	606685/2q32.2
TAZ	Tafazzin	DCM	X-linked	300394/ Xq28
TBX20	T-Box Transcription Factor 20	DCM	AD	606061/7p14.2
TCAP	Titin cap	DCM/HCM	AD	604488/17q12
TMEM43	Transmembrane Protein 43	DCM	AD	612048/3p25.1
TMPO/ LAP2	Thymopoietin	DCM	AD	188380/12p23.1
TNNC1	Troponin C1	DCM/HCM	AD	191040/3p21.1
TNNI3	Troponin I3	DCM/HCM/RCM	AD	191044/19q13.42
TNNT2	Troponin T2, cardiac	DCM/HCM/RCM	AD	191045/1q32.1
TPM1	Tropomyosin 1	DCM/HCM	AD	191010/15q22.2
TTN	Titin	DCM/HCM	AD	188840/2q31.2
VCL	Vinculin	DCM/HCM	AD	193065/10q22.2

AD: autosomal dominant; AR: autosomal recessive; X-linked: the gene causing the trait or the disorder is located on the X chromosome

Table 2. List of published genes associated with hypertrophic cardiomyopathy was used for screening variants in the study

Gene	Protein	Phenotype	Inheritance	OMIM/Chr
**HCM caused by genes encoding sarcomeric proteins**				
ACTC	Actin alpha cardiac	DCM/HCM	AD	102540/15q14
MYBPC3	Myosin binding protein 3	DCM/HCM	AD	600958/11p11.2
MYH6	Myosin heavy chain 6	DCM/HCM	AD	160710/14q11.2
MYH7	Myosin heavy chain 7	DCM/HCM/RCM	AD	160760/14q11.2
MYL2	myosin regulatory light chain	DCM/HCM	AD	160781/12q24.11
MYL3	myosin regulatory light chain	DCM/HCM	AD	160790/3p21.31
TCAP	Titin cap	DCM/HCM	AD	604488/17q12
TNNI3	Troponin I3	DCM/HCM/RCM	AD	191044/19q13.42
TNNT2	Troponin T2, cardiac	DCM/HCM/RCM	AD	191045/1q32.1
TPM1	Tropomyosin 1	DCM/HCM	AD	191010/15q22.2
TTN	Titin	DCM/HCM	AD	188840/2q31.2
**HCM caused by metabolic/infiltrative disease**				
GLA	a-galactosidase	HCM	X-linked	300644/Xq22.1
LAMP2	lysosome associated membrane protein 2	HCM	X-linked	309060/Xq24
PRAGK2	AMP-activated protein kinase subunit	HCM	AD	602743/7q36.1

AD: autosomal dominant; X-linked: the gene causing the trait or the disorder is located on the X chromosome
